# Starting and Operating a Public Cardiac Catheterization Laboratory in a Low Resource Setting: The Eight-Year Story of the Uganda Heart Institute Catheter Laboratory

**DOI:** 10.5334/gh.859

**Published:** 2021-02-09

**Authors:** Joselyn Rwebembera, Twalib Aliku, James Kayima, Sulaiman Lubega, Elias Sebatta, Brian Kiggundu, Daudi Kirenzi, Wilson Nyakoojo, Charles Mondo, Krishna Kumar, Kanishka Ratnayaka, Shakeel Qureshi, Sanjay Daluvoy, Peter Lwabi, John Omagino, Craig Sable, Chris Longenecker, Dan Simon, Marco Costa, Emmy Okello

**Affiliations:** 1 Uganda Heart Institute, Kampala, UG; 2 Makerere University, Kampala, UG; 3 Nsambya Hospital, Kampala, UG; 4 Amrita Institute of Medical Sciences, Kochi, IN; 5 Rady Children’s Hospital, San Diego California, US; 6 Evelina London Children’s Hospital, London, UK; 7 World Children’s Initiative, North Carolina, US; 8 The Children’s National Health System, Washington DC, US; 9 University Hospitals Cleveland Medical Center and Case Western Reserve University, Cleveland, Ohio, US

**Keywords:** cardiac, catheterization, laboratory, cath lab, Uganda, CVD, LMIC, Africa

## Abstract

**Background:**

Low- and-middle-income-countries (LMICs) currently bear 80% of the world’s cardiovascular disease (CVD) mortality burden. The same countries are underequipped to handle the disease burden due to critical shortage of resources. Functional cardiac catheterization laboratories (cath labs) are central in the diagnosis and management of CVDs. Yet, most LMICs, including Uganda, fall remarkably below the minimum recommended standards of cath lab:population ratio due to a host of factors including the start-up and recurring costs.

**Objectives:**

To review the performance, challenges and solutions employed, lessons learned, and projections for the future for a single cath lab that has been serving the Ugandan population of 40 million people in the past eight years.

**Methods:**

A retrospective review of the Uganda Heart Institute cath lab clinical database from 15 February 2012 to 31 December 2019 was performed.

**Results:**

In the initial two years, this cath lab was dependent on skills transfer camps by visiting expert teams, but currently, Ugandan resident specialists independently operate this lab. 3,542 adult and pediatric procedures were conducted in 8 years, including coronary angiograms and percutaneous coronary interventions, device implantations, valvuloplasties, and cardiac defect closures, among others. There was a consistent expansion of the spectrum of procedures conducted in this cath lab each year. The initial lack of technical expertise and sourcing for equipment, as well as the continual need for sundries present(ed) major roadblocks. Government support and leveraging existing multi-level collaborations has provided a platform for several solutions. Sustainability of cath lab services remains a significant challenge especially in relation to the high cost of sundries and other consumables amidst a limited budget.

**Conclusion:**

A practical example of how centers in LMIC can set up and sustain a public cardiac catheterization laboratory is presented. Government support, research, and training collaborations, if present, become invaluable leverage opportunities.

## Background

At the beginning of the 20th century, cardiovascular disease (CVD) was responsible for fewer than 10% of all deaths worldwide. Today, that figure is about 30%, with 80% of the burden now occurring in low- and middle-income countries (LMIC) [1,2,3,4,5]. Yet, LMIC, including Uganda, are underequipped to handle this burden in the setting of limited diagnostic and therapeutic equipment, limited availability of highly specialized personnel, constrained national budgets, and multiple competing national priorities, among other significant limitations [3]. These challenges partially explain the high premature mortality and morbidity in LMICs in relation to cardiovascular diseases [5]. Well-planned development, if sustained, can make enormous differences in the diagnosis, management, and outcomes of patients with cardiovascular diseases in these countries.

A spectrum of congenital and acquired cardiovascular disorders can be diagnosed and treated in the cardiac catheterization laboratory. These diseases include coronary artery disease, cardiac valve pathologies, several congenital cardiac abnormalities, and vascular disorders. An estimated 7.4 million of the 17.7 million CVD deaths in 2015 were due to coronary artery disease, with over three-quarters of these deaths taking place in LMICs [5]. In Uganda, for a population of about 42 million people with a population growth of 3.0% per year, it is estimated that annually about 10,000 children are born with congenital heart disease. Of these, 2,500 are severe enough to require cardiac interventions [6]. Rheumatic heart disease (RHD) is still the leading cause of cardiovascular mortality and morbidity among young adults in developing countries, including Uganda [7]. For example, rheumatic mitral valve stenosis, which is very poorly tolerated (particularly during pregnancy) and other conditions that exert stress on an individual’s hemodynamics, can often be treated in the cath lab.

**Figure 1:**
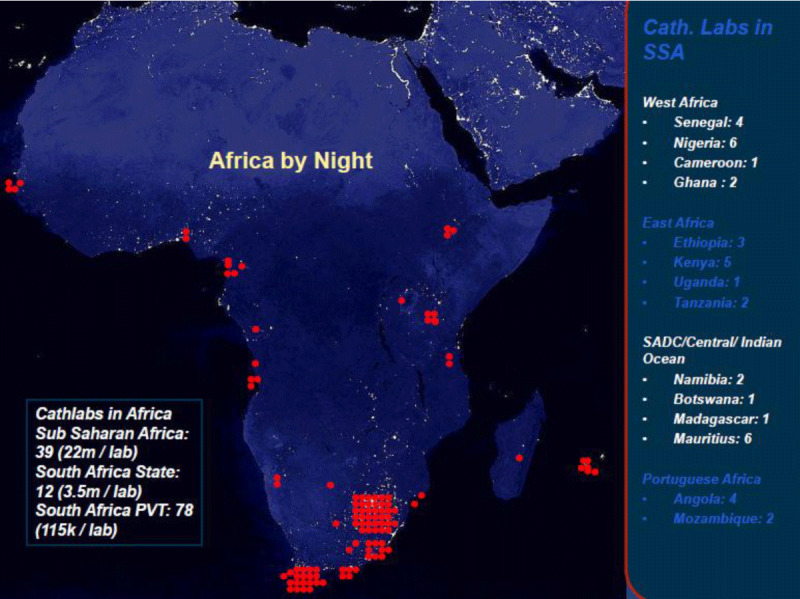
Number of Cardiac Catheterization laboratories in Sub-Saharan Africa in 2018. Adopted from Dr. Graham Cassel, Milpark Hospital, Johannesburg. The need and distribution of cath labs for adult and pediatric cases throughout Africa. https://www.tctmd.com/slide/distribution-cath-labs-throughout-africa.

Despite the central role that cardiac catheterization laboratories play in the diagnosis and management of CVDs, there is a critical shortage of these laboratories in sub-Saharan Africa, with the majority of countries falling remarkably below the minimum recommended standard of 1 cardiac catheterization laboratory per 1,000,000 population [8,9]. (Figure 1) Uganda has one operational cath lab out of the expected 40 cath labs following this recommendation. Installing and maintaining a cardiac catheterization laboratory is expensive because of the installation and maintenance costs of the equipment, the requirement of dedicated trained personnel, and the need to continually stock a large inventory of expensive consumables, both for diagnosis and interventions [10,11]. This, coupled with scarcity of highly specialized personnel with the technical expertise to perform and support procedures in the few existing laboratories, has greatly limited the installation of cardiac catheterization laboratories and performance of life saving procedures – despite the high burden of CVDs in the rapidly-growing populations in LMICs. The strategy of skills transfer camps, with visiting teams flying into developing countries once or twice a year to perform specialized procedures, is a helpful start. It offers training opportunities to a larger number of staff across multiple disciplines at an institution. Still, the impact of such a strategy on clinical care is limited [12,13] as these missions can treat only a few patients from lengthy waiting lists.

### The history of interventional cardiology in Uganda and the current study site

The first cardiac catheterization laboratory was installed in Mulago National Referral Hospital in 1960 and performed right and left heart diagnostic cardiac catheterizations. However, this service collapsed during the political turmoil of the 1970–1980s, and there were no invasive cardiac catheterization services until 2012, when the current catheterization laboratory was installed at UHI. This cath lab installation was completed on 12 April 2012, and the first procedure, a diagnostic coronary angiogram was performed on 30 April 2012, under the proctorship of Dr. Leonard Steingo from South Africa. The Uganda Heart Institute provides tertiary cardiovascular services for the whole of Uganda and a few neighboring countries. The country’s single cardiac catheterization laboratory serves a population of approximately 42 million children and adults. There are two pediatric and five adult interventional cardiologists at this center. Interventional and diagnostic cardiac catheterization procedures in the initial period (2012–2013) were conducted through a series of skills transfer camps with local cardiologists and technologists working under the supervision of experienced visiting teams. Subsequently, Ugandan personnel underwent training at different collaborating institutions [6,14]. (Table 1) Over time, a team of independent interventional cardiologists and technologists who routinely perform a variety of diagnostic and interventional procedures was built.

**Table 1:** Global institutions where personnel from UHI received training in interventional cardiology.

Collaborating Institution	Training Offered

Amrita Institute of Medical Sciences and Research, Kerala	Pediatric interventional cardiology
Case Western Reserve University – Ohio, USA	Clinical cardiology, adult interventional cardiology, advanced heart failure fellowships.
Frontier Lifeline Hospital – Chennai, India	Clinical cardiology, cardiac electrophysiology
Madras Medical Mission – Chennai, India	Clinical and interventional cardiology
Malabar Institute of Medical Sciences, India	Catheterization laboratory technical training
Medanta Hospital – New Delhi, India	Clinical and interventional cardiology
National Heart Institute – Kuala Lumpur, Malaysia	Catheterization laboratory technical training
University of Cape Town – South Africa	Clinical and interventional cardiology

The objective of this study is to review the eight-year journey of running a single cardiac catheterization laboratory in Uganda, while paying particular attention to the procedures performed, the lessons learned, the challenges surmounted, and the projections for the future.

## Methods

### Study design

A retrospective review of the Uganda Heart Institute cardiac catheterization laboratory clinical database from 15 February 2012 to 31 December 2019 was performed.

### Study site

The Uganda Heart Institute is a specialized tertiary center for cardiovascular care in Uganda located within Mulago Hospital Complex, the country’s national referral hospital and teaching hospital for Makerere University Medical School. The country’s single cardiac catheterization laboratory is a fully functional Siemens Artis Zee bi-plane with Syngo Software and Sensis Hemodynamic system which was installed in April 2012. The adult and paediatric UHI out-patient and in-patient departments offer support to the catheterisation laboratory. Annually, UHI handles approximately 20,000 OPD consultations, 12,000 in-patient admissions, 22,000 transthoracic echocardiograms, 25,000 resting electrocardiograms, and 3,000 other procedures including exercise electrocardiograms, Holters and trans-eosophageal echocardiograms.

### Study population

The study population consisted of consecutive children and adults who had a diagnostic and/or interventional procedure performed in the cardiac catheterization laboratory during the study period. Retrospective database review was conducted.

### Diagnostic cardiac catheterization and interventional procedures

The UHI cath lab follows current guidelines and standards set by the American Cardiology of Cardiology and Society for Cardiovascular Angiography and Imaging [15,16].

### Data management

Data were initially captured in Epi Info 3.0 and later exported to Stata 12.0. Categorical variables are presented as count (percentage), while continuous variables are expressed as means ± standard deviation. This is the inaugural publication under the auspices of the Uganda Heart Institute Cardiac Catheterisation Registry: Patient profile, procedural complications, and long-term outcomes (#REC REF 2020–190). Individual patient informed consent was not required.

## Results

All 3,542 procedures performed during the study period were included in the data analysis. (Table 2) These procedures are classified into those performed by the adult interventional cardiologists (2,916 procedures) and those performed by the pediatric interventional cardiologists (626 procedures). Diagnostic coronary angiographies accounted for 65% of procedures performed by the adult interventional team, while PDA device closures formed the bulk (50%) of procedures performed by the pediatric interventional team.

**Table 2:** Total number of procedures performed during the study period.

Procedure	Total No. of Procedures, 2012–2019

Procedures Performed By The ‘Adult Interventional’ Team; N = 2,916	

Coronary angiography	1,902 (65%)
Percutaneous coronary interventions	408 (14%)
Single & dual chamber pacemaker implantation	246 (8%)
Temporary pacemaker insertion	166 (5.7%)
Cardiac resynchronization therapy	61 (2.1%)
ICD implantation	3 (0.1%)
Balloon mitral valvuloplasty	81 (2.8%)
Electrophysiology studies +/– RFA	24 (0.8%)
IVC filter insertion	13 (0.4%)
Peripheral angiography	4 (0.1%)
Intra-aortic balloon pump (IABP) placement	3 (0.1%)
Endovascular aortic repair	5 (0.2%)
**Total**	**2,916 (100%)**
**Procedures Performed By The ‘Paediatric Interventional’ Team; N = 626**	

PDA device closure	317 (50.6%)
Balloon pulmonic valvuloplasty	77 (12.3%)
Diagnostic catheterization	180 (28.8%)
Balloon atrial septostomy	19 (3.0%)
ASD device closure	8 (1.3%)
VSD device closure	7 (1.1%)
Others	18 (2.9%)
**Total**	**626 (100%)**

ICD: Implantable Cardiac Defibrillator; RFA: Radio-Frequency Ablation; IVC: Inferior Vena Cava; IABP: Intra-Aortic Balloon Pump; PDA: Patent Ductus Arteriosus; ASD: Atrial Septal Defect; VSD: Ventricular Septal Defect.

### Timeline of diagnostic and interventional cardiac procedures in the cardiac catheterization laboratory at Uganda Heart Institute

**Figure 2:**
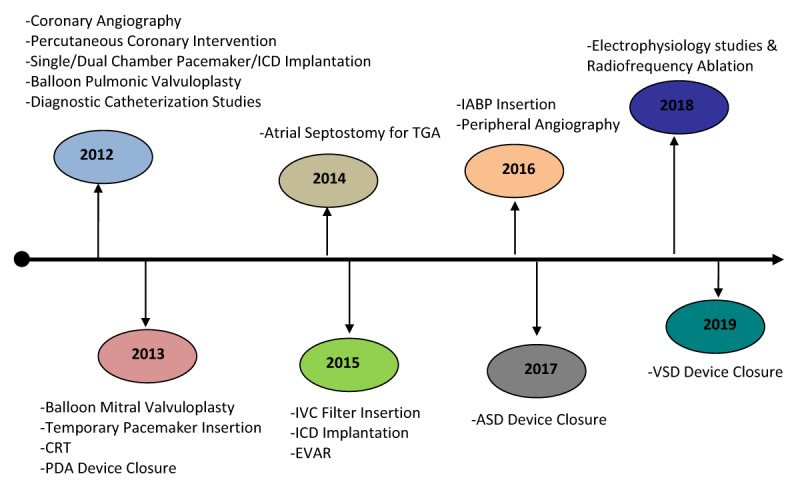
Evolution of invasive diagnostic and interventional cardiac procedures in the cardiac catheterization laboratory of Uganda Heart Institute. ICD: Implantable Cardiovertor Defibrillator; CRT: Cardiac Resynchronization Therapy; PDA: Patent Ductus Arteriosus; TGA: Transposition of the Great Arteries; IVC: Inferior Vena Cava; EVAR: Endovascular Aortic Repair; IABP: Intra-Aortic Balloon Pump; ASD: Atrial Septal Defect; VSD: Ventricular Septal Defect.

There has been a steady evolution of diagnostic and interventional cardiac procedures in the cardiac catheterization laboratory. (Figure 2) The earliest procedures to be performed in 2012 included coronary angiography, percutaneous coronary intervention, single and dual chamber pacemaker insertion, balloon pulmonic valvuloplasty, and right and left heart diagnostic catheterizations; the latest addition in 2019 was ventricular septal defect (VSD) device closure.

### Number of procedures in each year: 2012–2019

**Figure 3:**
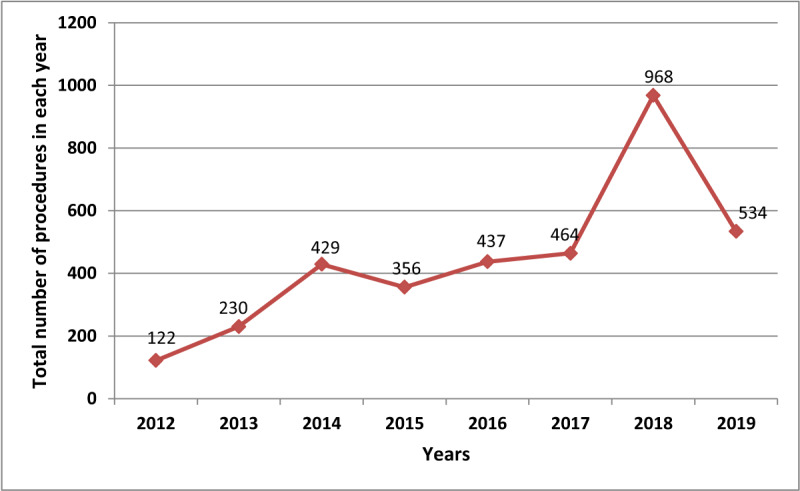
Total number of diagnostic and interventional procedures in each year, 2012–2019.

**Figure 4:**
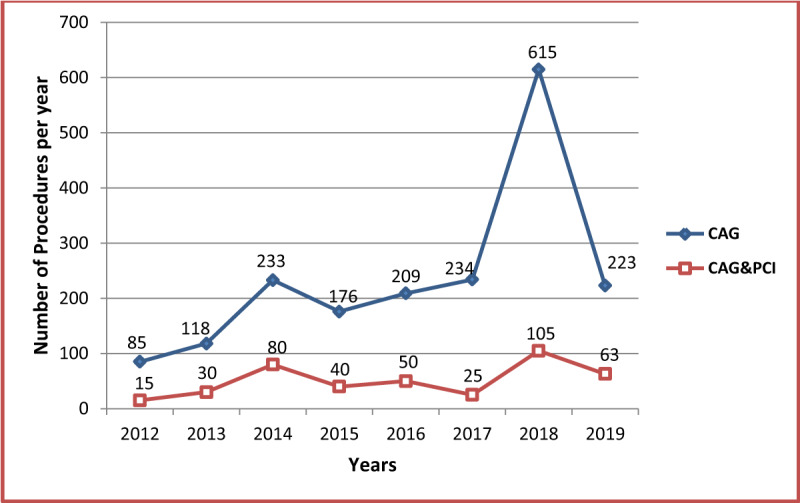
Number of diagnostic coronary angiography and percutaneous coronary intervention procedures in each year. CAG: Coronary Angiography; PCI: Percutaneous Coronary Intervention.

There has been a progressive increase in the total number of procedures performed in each year since the installation of the laboratory. (Figure 3) Coronary artery procedures, including diagnostic coronary angiography and percutaneous coronary intervention (Figure 4), concomitantly peaked in 2018 when the number of procedures more than doubled the number of the preceding year. The implantation of single and dual chamber pacemakers peaked in 2016 and has remained constant in the subsequent years. The number of CRT and ICD implantation procedures has remained low and stayed within similar range over the years.

**Figure 5:**
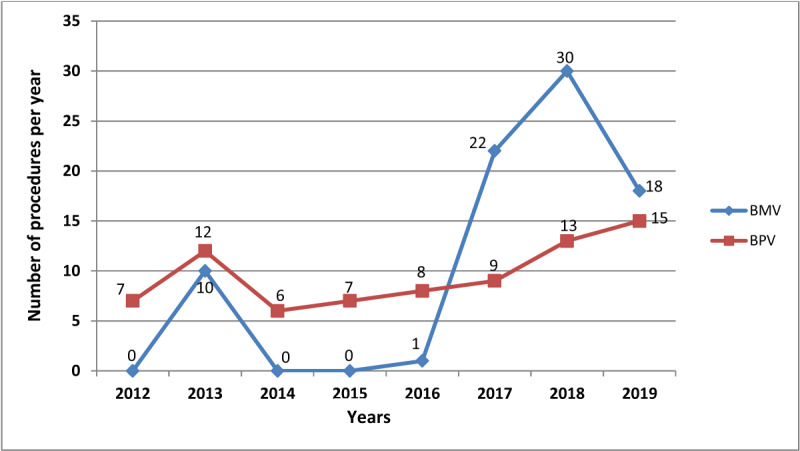
Number of balloon valvuloplasty procedures in each year. BMV: Balloon Mitral Valvuloplasty; BPV: Balloon Pulmonic Valvuloplasty.

Balloon mitral valvuloplasty is performed for rheumatic mitral stenosis. The initial ten procedures in 2013 were performed with a visiting team. Lack of resident team expertise in the procedure led to inactivity over the ensuing 3 years. However, following training of two adult interventional cardiologists at University Hospitals Cleveland Medical Center in Cleveland, USA – through a collaboration between Case Western Reserve University, Uganda Heart Institute, and Makerere University – the performance of BMV procedures purely by the resident team started in 2017. Details of this collaboration have been published previously [14]. The highest number of 30 procedures was registered in 2018. (Figure 5) Balloon pulmonic valvuloplasty is performed for pulmonic valve stenosis by the pediatric interventional team, and patients include children and adults of all ages.

**Figure 6:**
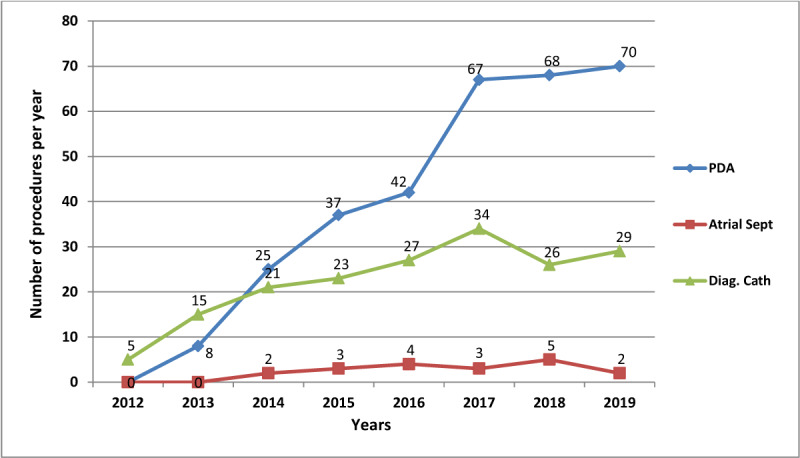
Number of patent ductus arteriosus device closures, atrial septostomies for TGA and diagnostic right and left heart catheterization procedures. PDA: Patent Ductus Arteriosus; Atrial Sept: Atrial Septostomy for TGA; Diag. Cath: Diagnostic right and left cardiac catheterization.

Device closure of patent ductus arteriosus started in 2012 when the catheterization laboratory was installed. (Figure 6) It is the procedure most frequently performed by the paediatric interventional team, and the number of procedures has consistently risen over the years, with a peak and stability in the past 3 years. Diagnostic right and left heart catheterization are also performed by the paediatric interventional team, but patients include children and adults of variable ages. This is the second most frequently performed procedure by the paediatric team. Also having started in 2012, there has been a slow but steady increase in the annual procedures. (Figure 6) Atrial septostomies for transposition of the great arteries (TGA) have remained an occasional procedure over the years.

A small number of other procedures including electrophysiology studies +/– radiofrequency ablation, peripheral angiography, intra-aortic balloon insertion, inferior vena cava filter insertion, and endovascular aortic repair have been intermittently performed. Table 3 shows the number of these and the cardiac pacing procedures that have been performed in each year.

**Table 3:** Number of procedures that have been performed intermittently since installation of the cardiac catheterization laboratory.

Procedures	Years							

	2012	2013	2014	2015	2016	2017	2018	2019

Permanent Pacemaker Insertion (Single & Dual Chamber)	7	18	37	30	40	38	36	40
Temporary Pacemaker Insertion	0	12	10	25	34	15	30	40
Cardiac Resynchronization Therapy (CRT-D & CRT-P)	0	4	12	10	12	5	9	9
ICD Implantation	0	0	0	1	1	0	0	1
EPS/RFA	0	0	0	0	0	0	14	10
Peripheral Angiography	0	0	0	0	4	0	0	0
Intra-Aortic Balloon Pump Insertion	0	0	0	0	2	0	1	0
IVC Filter Insertion	0	0	0	2	1	1	5	4
EVAR	0	0	0	1	1	1	2	0

EPS: Electrophysiology Studies; RFA: Radiofrequency Ablation; IVC: Inferior Vena Cava; EVAR: Endo-Vascular Aortic Repair.

### Immediate procedure outcomes

Out of the 3,542 procedures, there were eight deaths within the cath lab, five of which were related to the severity of the medical condition (extensive ST elevation myocardial infarction with haemodynamic and electrical instability) rather than direct procedural complications. There were three transfers to open heart surgery following acute complications of cardiac catheterisation and intervention. This is an immediate, intra-cath lab serious complication rate of < 0.5%.

### Financing of procedures

Of the 626 paediatric procedures, approximately 70% were financed through philanthropic support. The major philanthropic players have included, but are not limited to: Gift Of Life; Rotary International and Chain of Hope. Several Ugandan non-governmental organisations and well-wishers have also sponsored a bulk of procedures. The other 25% were facilitated by out-of-pocket payments by families while 5% were by health insurance coverage. Notably, there is evident increase in the family- and insurance-facilitated procedures in the past 2–3 years. For the 2,916 adult procedures, approximately 98% were financed through out-of-pocket payments by families, while 2% were facilitated by health insurance coverage. The out-of-pocket payment system for both the paediatric and adult procedures is a co-pay system whereby the government, through votes to UHI, finances 50% of the procedure costs while the patients and their families pay 50%. Therefore, the upfront cost that the families must pay is significantly subsidized. Pacemaker devices are paid for fully and directly from the in-country representatives of pacemaker manufacturers at the running market price, while the consumables that are used for the implantation procedures are financed through the government-patient co-pay system.

### Growth rate of the cardiac catherization laboratory

The annual growth rates by number of annual procedures were 88% (2012–2013); 86% (2013–2014); –17% (2014–2015); 23% (2015–2016); 6% (2016–2017); 108% (2017–2018); and –45% (2018–2019). The average growth rate was 35% per annum.

## Discussion

This paper reviews and presents data on the evolution of cardiac catheterization in Uganda, a single public-owned facility in the country. Development of local technical expertise through training and research collaborations, and government support is reviewed. Performance of high quality invasive and interventional cardiology with the highest levels of safety was the goal for installation of the catheterization laboratory. The needs of the patient, the government (which funds the Uganda Heart Institute), and the physicians across the country needed to be met in order to achieve this goal.

### Overall performance

Over 3000 procedures were performed in the study period, including both paediatric and adult populations. Seven hundred cases done between 2012 and 2014 were performed during skills transfer camps by experts from centers that already had established cardiac catheterization. Proctors came from centers in South Africa, India, Kenya, the United States of America, Italy, and the United Kingdom. During this time, Ugandan cardiologists started formal interval training in interventional cardiology. (Table 1) In 2014, the first formally trained interventional cardiologists returned, which accounts for the sudden increase in the number of procedures done, as several adult and paediatric cases were performed daily.

Our paediatric and adult interventional programs have been supportive of each other with the adult interventional team performing some procedures in the paediatric population (e.g., mitral valvuloplasty); while the paediatric interventional team has performed procedures in the adult population (e.g., pulmonic valvuloplasty). This proves that when there is effective adult-paediatric collaboration, these services can grow together and are not competitors [6,14].

The immediate intraprocedural complication rate is similar to that which is observed even in the developed settings [15,16]. The concurrent establishment and growth of the cardiothoracic surgery service at UHI has provided invaluable support to the continuity of the cath lab. Resident cardiac surgeons have availed back-up for all interventional procedures, and have adequately intervened in the < 0.5% cases of cardiac catheterization procedural complications. After this inaugural publication, procedure-specific analyses and publications will be performed, where short- and long-term outcomes will be reported.

This example emphasizes the importance of the tripartite academic mission – clinical care, research, and education. Grant-supported research and personnel training have been the springboards for the sustenance of this cath lab service [14].

### The role of the cardiac catheterization laboratory in ‘tropical cardiology’

Despite the emergence of CVDs of affluence, like coronary artery disease, endemic CVDs like RHD in our environment still require attention and can benefit from interventional procedures. BMV is a lifesaving procedure, especially in states such as pregnancy. As the scope of procedures performed in this cath lab are expanded, TAVR is envisioned as a future possibility. It is yet to be known whether TAVR can be safely and effectively performed in RHD. If it proves a viable option in the future, it shall be embraced for suitable RHD patients in this setting. The parallel development of cardiothoracic surgery at this institution has been complementary to the cath lab services particularly in scenarios of failed or complicated interventional procedures.

### Challenges and solutions

Several challenges stood in the way of the newly installed cardiac catheterization laboratory including: lack of technical expertise, lack of trained interventional cardiologists, catheterization laboratory support staff and sourcing for the catheterization laboratory sundries and equipment. With government support, the leadership of UHI was able to leverage expertise from existing collaborations from Children’s National Health System (Washington DC) and subsequently World Children’s Initiative (WCI; www.wciprojects.org) to guide the design and choice of the cardiac catheterization laboratory equipment and subsequent sundries.

Planning for the cardiac catheterization laboratory required support of the government to provide for it in the annual budget. The Ugandan government also gave special procurement considerations due to the unique nature of the equipment and its process of manufacture.

World Health Organization building blocks of health systems were followed, which include health service delivery; health workforce; health information systems; access to essential medicines; health systems financing; and leadership and governance [17] in making annual plans and evaluating the performance of the cath lab.

Human resource has always been a significant challenge and focus of efforts. A gradual plan for staff training started with cardiac catheterization laboratory nurses and technicians being trained at centers in India and Malaysia. This was followed by training of interventional cardiologists in India, the United States, and South Africa. (Table 1) The UHI leveraged training and research collaboration involving Makerere University and Case Western Reserve University that supported the research component of the Uganda Rheumatic Heart Disease registry. Makerere University, through a MEPI-CVD linked award, supported Ugandan cardiologists’ training in interventional cardiology in Cleveland, Ohio. This allowed both the research program and the training to grow exponentially. This training eventually expanded into heart failure and cardiac anesthesia [14]. The paediatric interventional cardiology training has been supported over the years through a collaboration with WCI.

The lack of back-up equipment has created gaps in service delivery. For example, after a steep increase in the overall number of procedures in 2018 was realized, there was an almost-equally sharp fall in 2019 due to cath lab down time of close to 4 months in that year. Being the only cath lab serving a large population it was subject to wear and tear. At the same time, it was necessary to rely on biomedical teams from Nairobi, Kenya to service and repair the equipment.

Today, UHI is in the final stages of procuring and installing a second catheterisation laboratory which is meant to allow more procedures while concurrently reducing the burden of the current laboratory. Also, now in-country are cath lab biomedical engineers who are able to attend to any equipment malfunctions in a prompt and timely manner. Despite the government’s contribution to the cost of procedures, many patients are still unable to afford the subsidized prices and therefore remain on conservative medical management. The most viable solution to this is a widespread national health insurance scheme. The patients who reach UHI are only the tip of an iceberg of the cardiovascular disease burden in Uganda. There is need to decentralize specialized cardiology services to regional and district hospitals to extend specialized diagnostic and therapeutic services to all strata of socioeconomic classes in the country. There also remains gross underutilisation of some available equipment. For example, the intra-aortic balloon pump has only been used occasionally despite the many deserving patients due to the hefty cost of consumables.

With an average annual growth rate of 35% amidst multiple challenges, faster growth and a broader spectrum of procedures are anticipated over the next decade particularly with the forthcoming installation of more cath labs. Two cath labs are being installed in private hospitals within the city of Kampala and should be operational in a space of a few months. These will ease the burden on the current single operational cath lab in the country.

Despite the challenges faced, the presence of this cath lab has spurred cardiovascular care growth in Uganda by availing more treatment options, more training and research while concurrently reducing out-of-country medical referrals. The Uganda Heart Association, through a U-STEMI (Uganda-acute ST segment elevation myocardial infarction) network, has created a hub-and-spoke model with UHI being the hub and other large city and regional hospitals being the spokes. Through training on recognition of cardiac emergencies, particularly acute coronary syndromes – severe bradycardia among others – there has been increased awareness, and therefore increased referrals, of patients from across the country but most especially from the city hospitals.

### Training the next generation of skilled personnel at UHI

The Uganda Heart Institute has established a cardiology fellowship training program since 2013. Residents/registrars qualifying in internal medicine, paediatrics, surgery, and anesthesia enroll for 3-year training programmes in adult cardiology, paediatric cardiology, cardiac surgery, and cardiac anaesthesia. A total of 15 cardiology specialists have graduated from this programme, all of whom are working in Uganda. Rotation in the cardiac catheterization laboratory is mandatory during the fellowship training. Some fellows have gained interest in interventional cardiology and are undergoing further, specialized training.

## Limitations

The numbers of procedures in this manuscript do not portray the number of persons in Uganda who require these procedures. These numbers reflect only the patients that were able to reach UHI and afford the service, and are most likely a gross underestimation of the burden of cardiovascular disease in the country.

## Conclusion

This cath lab is a practical example of how centers in LMIC can set up a public cardiac catheterization laboratory. Government support, research, and training collaborations, if present, become invaluable leverage opportunities.

## Abbreviations

LMIC: Low- and Middle-Income Countries

CVD: Cardiovascular Disease

RHD: Rheumatic Heart Disease

UHI: Uganda Heart Institute

PDA: Patent Ductus Arteriosus

ICD: Implantable Cardioverter Defibrillator

RFA: Radiofrequency Ablation

IVC: Inferior Vena Cava

IABP: Intra-Aortic Balloon Pump

ASD: Atrial Septal Defect

VSD: Ventricular Septal Defect

CRT: Cardiac Resynchronization Therapy

TGA: Transposition of the Great Arteries

EVAR: Endovascular Aortic Repair

CAG: Coronary Angiogram

PCI: Percutaneous Coronary Intervention

BMV: Balloon Mitral Valvuloplasty

BPV: Balloon Pulmonic Valvuloplasty
